# *BMC Ophthalmology* reviewer acknowledgement 2015

**DOI:** 10.1186/s12886-016-0193-5

**Published:** 2016-02-11

**Authors:** Anna Clark

**Affiliations:** BioMed Central, Floor 6, 236 Gray’s Inn Road, London, WC1X 8HB UK

## Abstract

The editors of *BMC Ophthalmology* would like to thank all our reviewers who have contributed to the journal in Volume 15 (2015).

**Mohammed Abdull**

Nigeria

**Massimo Accorinti**

Italy

**James Acheson**

UK

**Bola Adekoya**

Nigeria

**Srijana Adhikari**

Nepal

**Rupesh Agrawal**

Singapore

**Zubair M. Ahmed**

USA

**Mehmet Orcun Akdemir**

Turkey

**Sibel Aksoy**

Turkey

**Luciana Alencar**

Brazil

**Mohammad Ali**

India

**Jorge Alio**

Spain

**Micol Alkabes**

Italy

**Ryham Allam**

Egypt

**Benjamin Althouse**

USA

**Winfried Amoaku**

UK

**Afsaneh Amouzegar**

USA

**Kumarasamy Anbarasu**

India

**Marcus Ang**

Singapore

**Rajendra Apte**

USA

**James Aquavella**

USA

**Maria Cecilia Aquino**

USA

**Ilir Arapi**

Italy

**Yonca Arat**

Turkey

**Samuel Arba-Mosquera**

Spain

**Luis Arias**

Spain

**Murali Ariga**

India

**Mary Beth Aronow**

USA

**Hemamalini Arvind**

Australia

**Penny Asbell**

USA

**George Asimellis**

USA

**Ioannis Aslanides**

Greece

**Rashima Asokan**

India

**Marcel Avila**

Colombia

**Ahmed Awadein**

Egypt

**Marcelo Ayala**

Sweden

**Kalpana Babu Murthy**

India

**Simon Backhouse**

Australia

**Jeong Hun Bae**

South Korea

**Haiqing Bai**

China

**Konstantinos Balaskas**

UK

**Brendan Barrett**

UK

**Robert Barry**

UK

**Daniel Barthelmes**

Switzerland

**Keith Barton**

UK

**Ken Bassett**

Canada

**Roberto Bellucci**

Italy

**Pooja Bhat**

USA

**Muna Bhende**

India

**Marc Biarnés**

Spain

**Catherine Birt**

USA

**Jenefer Blackwell**

Australia

**Maria Antonietta Blasi**

Italy

**ostas Boboridis**

Greece

**Kathryn Bollinger**

USA

**Vincenza Bonfiglio**

Italy

**Nacim Bouheraoua**

France

**Banu Bozkurt**

Turkey

**John Bradbury**

UK

**Christopher Brand**

UK

**Milam Brantley**

USA

**Fion Bremner**

UK

**Dominique Bremond-Gignac**

France

**Scott Brodie**

USA

**Paolo Brusini**

Italy

**Vatinee Bunya**

USA

**Kathryn Burdon**

Australia

**Suk Ho Byeon**

South Korea

**Florence Cabot**

USA

**Delia Cabrera Debuc**

USA

**Pilar Cacho-Martínez**

Spain

**Halil Çagatay**

Turkey

**Pilar Calvo**

Spain

**Charles Campbell**

USA

**Gonzalo Carracedo**

Spain

**Ester Carreño**

UK

**Pilar Casas-Llera**

Spain

**Laure Caspers**

Belgium

**Heidi Cate**

UK

**Lingping Cen**

China

**Subhabrata Chakrabarti**

India

**Patricia Chalela**

USA

**Spyridon Chalkiadakis**

UK

**Jagdish Chander**

India

**Jessica Chang**

USA

**Shu-Hong Chang**

USA

**Neil Charman**

UK

**William Charman**

UK

**Sunil Chauhan**

USA

**Celia Chen**

Australia

**Chian-Feng Chen**

Taiwan

**Haoyu Chen**

China

**Hsin-Yi Chen**

Taiwan

**Philip Chen**

USA

**Connie Chen**

USA

**Andy Cheng**

Hong Kong

**Wah Cheuk**

Hong Kong

**Carol Cheung**

Singapore

**Shravan Chintala**

USA

**Lidia Chomicz**

Poland

**Kam-Lung Kelvin Chong**

Hong Kong

**Nikhil Choudhari**

India

**Bonnie Nga Kwan Choy**

Hong Kong

**cqueline Chua**

Singapore

**Jin Kwon Chung**

South Korea

**Tae-Young Chung**

South Korea

**Gerry Clare**

UK

**Rosa M. Coco**

Spain

**Cris Constantinescu**

UK

**Maria Cordeiro**

UK

**Luis Cordoves**

Spain

**Terry Coursey**

USA

**Margaux Crabtree**

USA

**Jennifer Craig**

New Zealand

**Ian Cree**

UK

**J. Oscar Croxatto**

Argentina

**Yan Cui**

China

**Arthur Cummings**

Ireland

**Tanuj Dada**

India

**Erika Damato**

UK

**Vivek Dave**

India

**Helen Davis**

UK

**Janet Davis**

USA

**Roberto Dell'Omo**

Italy

**Hakan Demirci**

USA

**Alastair Denniston**

UK

**Maria Dettoraki**

Greece

**Christoph Deuter**

Germany

**Federico Di Matteo**

Italy

**Vasilios Diakonis**

USA

**Xiaoyan Ding**

China

**Ali Djalilian**

USA

**Rosa Dolz-Marco**

Spain

**Rodrigo Donoso**

Chile

**Stella Douma**

Greece

**Laura Downie**

Australia

**Alina Dumitrescu**

USA

**Yusuf Durlu**

Turkey

**Matthew Edmunds**

UK

**Rita Ehrlich**

Israel

**Amal Elbendary**

Egypt

**Mostafa Elgohary**

UK

**Hala Elhilali**

Egypt

**Charles Elmaraghy**

USA

**Gehad Elnahri**

Egypt

**Rasha Eltanamly**

Egypt

**Gabriela Espinoza**

USA

**Boniface Ikenna Eze**

Nigeria

**Mads Krüger Falk**

Denmark

**Sepehr Feizi**

Iran

**Maryam Ferdousi**

UK

**Karen D. Fern**

USA

**Merle Fernandes**

India

**Roberto Fernandez Buenaga**

Spain

**Giulio Ferrari**

Italy

**Tiago Ferreira**

Portugal

**Alistair Fielder**

UK

**Michele Figus**

Italy

**Robert Finger**

Australia

**John Flatter**

USA

**Ignacio Flores-Moreno**

Spain

**Paolo Fogagnolo**

Italy

**Alex Fonollosa**

Spain

**Alejandro Fonollosa Calduch**

Spain

**Clare Fraser**

USA

**Valeria Fu**

USA

**Emre Güler**

Turkey

**Subhash Gaddipati**

USA

**Roberto Gallego-Pinazo**

Spain

**Juan Eduardo Gallo**

Argentina

**Nicola Gan**

Singapore

**Alfredo Garcia-Layana**

Spain

**Yonathan Garfias**

Mexico

**Sumit Garg**

USA

**David Gaucher**

France

**Bruce Gaynes**

USA

**Yubin Ge**

USA

**Ronnie George**

India

**Elham Ghahari**

USA

**Asaad Ghanem**

Egypt

**Khalil Ghasemi Falavarjani**

Iran

**Stephen Gichuhi**

UK

**Ilene Gipson**

USA

**Fleur Goezinne**

Netherlands

**E-Shawn Goh**

Singapore

**Kong Yong Goh**

Singapore

**Francisco Javier Goñi**

Spain

**Johannes Gonnermann**

Germany

**Michael Grentzelos**

Greece

**Ahmet Kaan Gunduz**

Turkey

**Huanqing Guo**

Ireland

**Noopur Gupta**

India

**Sang Beom Han**

South Korea

**Fiona Harris**

UK

**Jiucheng He**

USA

**Mark Heaney**

USA

**Anders Heijl**

Sweden

**Francisco Hernandez-Torres**

Spain

**Nicole Heussen**

Germany

**Taichi Hikichi**

Japan

**David Hinkle**

USA

**Nino Hirnschall**

Austria

**Kazuyuki Hirooka**

Japan

**Jesper Hjortdal**

Denmark

**Stephan Hoffmann**

Germany

**Vladimir Holan**

Czech Republic

**Gabor Hollo**

Hungary

**Shigeru Honda**

Japan

**Shinataro Horie**

Japan

**Anna Horwood**

UK

**Akihiko Hoshi**

Japan

**Mohsen Hosseini**

Canada

**Yu-Chih Hou**

Taiwan

**Yi-Hsun Huang**

Taiwan

**Ruth Huna-Baron**

Israel

**Toby Hurd**

UK

**Michele Iester**

Italy

**Makoto Inoue**

Japan

**Makoto Ishikawa**

Japan

**J. Iyer**

Singapore

**Gregory Jackson**

USA

**Robert Jacobs**

New Zealand

**Mohammadreza Jafarinasab**

Iran

**Ebrahim Jafarzadehpur**

Iran

**Marilyn James**

UK

**Sun Young Jang**

South Korea

**Vishal Jhanji**

Hong Kong

**Zhengxuan Jiang**

China

**Amit Jinabhai**

UK

**Jasleen Jolly**

UK

**Denis Jusufbegovic**

USA

**Andreas K. Lauer**

USA

**Namir Kafil-Hussain**

UK

**Alon Kahana**

USA

**Rim Kahloun**

Tunisia

**Swathi Kaliki**

India

**Yogita Kanan**

USA

**Hyong Kwon Kang**

Australia

**Hae Min Kang**

South Korea

**Chitra Kannabiran**

India

**Costas H. Karabatsas**

Greece

**Dimitrios Kardaras**

Greece

**Marzieh Katibeh**

Iran

**Andreas Katsanos**

Greece

**S Kavitha**

India

**Takahiro Kawaji**

Japan

**Oonuma Kazuhiko**

Japan

**Vladimir Kefalov**

USA

**Hamid Khakshoor**

Iran

**Mayuri Khamar**

India

**Jane Khan**

Australia

**Hemant Khanna**

USA

**Rohit Khanna**

India

**Nora Khatib**

USA

**Ahmad Kheirkhah**

USA

**Kyeong Hwan Kim**

South Korea

**Chan Yun Kim**

South Korea

**Tae Wan Kim**

South Korea

**Kenichi Kimoto**

Japan

**Ashwini Kini**

India

**Aman Kirmani**

UK

**Tomas Knapen**

Netherlands

**Konrad Koch**

Germany

**Triantafyllia Koletsa**

Greece

**Nataliya Kolosova**

Russia

**Georgios Kontadakis**

Greece

**Karanjit Kooner**

USA

**Dara Koozekanani**

USA

**Larry Koreen**

USA

**Rhonda G. Kost**

USA

**Mihir Kothari**

India

**Prabhakar Krishnacharya**

India

**David Krizaj**

USA

**KazuyUKi Kumagai**

Japan

**Vinod Kumar**

India

**Periyasamy Kumar**

UK

**Ajay Kumar**

USA

**James Kundart**

USA

**Shoji Kuriyama**

Japan

**Chiara La Morgia**

Italy

**Neil Lagali**

Sweden

**Jimmy Lai**

Hong Kong

**Timothy Lai**

Hong Kong

**Cedric Lamirel**

France

**Jonathan Lass**

USA

**Alex Lau**

Singapore

**Augustinus Laude**

Singapore

**Chang Kyu Lee**

South Korea

**Helena Lee**

UK

**Salomé Leibundgut**

Switzerland

**Gary Joseph Lelli**

USA

**Gary Lelli, Jr.**

USA

**Michael Lemp**

USA

**Kim Son Lett**

UK

**Richard Alan Lewis**

USA

**Monique Leys**

USA

**Qingfeng Liang**

China

**Xiaofeng Lin**

China

**Gopal Lingam**

Singapore

**Petra Liskova**

Czech Republic

**Julie-Anne Little**

UK

**Yongji Liu**

China

**Bingqian Liu**

China

**Wenzhong Liu**

USA

**Norberto Lopez-Gil**

Spain

**Yi Lu**

China

**Kenneth Lu**

USA

**Fiona Luk**

Hong Kong

**Jose Domingo Luna**

Argentina

**Li Ma**

Hong Kong

**Michele Madigan**

USA

**Dimitra Makrynioti**

Greece

**Alex Mammen**

USA

**Raffaele Mancino**

Italy

**Baskaran Mani**

Singapore

**Gianluca Manni**

Italy

**Ahmad Mansour**

Lebanon

**Kaweh Mansouri**

Switzerland

**Sanjay Marasini**

New Zealand

**Robert Marie-Claude**

Canada

**Inês Marques**

Portugal

**Marvin Marti**

Switzerland

**Enrico Martini**

Italy

**Vicente Martin-Montañez**

Spain

**Sundari Mase**

USA

**Ioannis Mavrikakis**

UK

**Julie Mcclelland**

UK

**Timothy Mcculley**

USA

**Paul Mcdaniel**

USA

**Rebecca Mcgreal**

USA

**Martin Mckibbin**

UK

**Rebecca Mclean**

UK

**Hemal Mehta**

USA

**Balraj Menon**

USA

**Farhan Merali**

USA

**Shannath Merbs**

USA

**Catherine Meyerle**

USA

**Manuele Michelessi**

Italy

**Atsuya Miki**

Japan

**Giovanni Milano**

Italy

**Dan Milea**

France

**Baruch Minke**

Israel

**Vikas Mittal**

India

**Garima Mittal**

India

**Sandra Mmontezuma**

USA

**Rahul Modak**

India

**Shaheeda Mohamed**

Hong Kong

**Aditi Mohla**

UK

**Gregory Moloney**

Australia

**Lieve Moons**

Belgium

**Bruce Moore**

USA

**Eduardo Moragrega**

Mexico

**Jiten Morarji**

UK

**Javier Moreno-Montañes**

Spain

**Nigel Morlet**

Australia

**Jim Morton**

New Zealand

**Majid Moshirfar**

USA

**Majid Moshirfar**

USA

**Lisha Mou**

China

**Guoying Mu**

Chile

**Kaustubh Mulay**

India

**Kosta Mumcuoglu**

Israel

**DaisUKe Muramatsu**

Japan

**Hiroshi Murata**

Japan

**Ian Murdoch**

UK

**Hemanth Murthy**

India

**K. Naeser**

Denmark

**Mayuresh Naik**

India

**Akira Nakai**

Japan

**Masatsugu Nakamura**

Japan

**HiroyUKi Namba**

Japan

**Mayank Nanavaty**

UK

**Raja Narayanan**

India

**Masood Naseripour**

Iran

**Neema Nayeb-Hashemi**

USA

**Paula Anne Newman-Casey**

USA

**Angeline Nguyen**

USA

**Simona Delia Nicoara**

Romania

**Juan Carlos Nieto**

Spain

**Kanwal Nischal**

USA

**Jason Noble**

Canada

**Benjamín Nogueda-Torres**

Mexico

**Monisha Esther Nongpiur**

Singapore

**Jean Jacques Noubiap**

Cameroon

**Katarzyna Nowomiejska**

Poland

**Carlo Nucci**

Italy

**William Nunery**

USA

**Jacqueline Nuysink**

Netherlands

**Anna O'Connor**

UK

**Veronica O'Dwyer**

Ireland

**Joo Youn Oh**

South Korea

**Toshihiko Ohta**

Japan

**Akio Oishi**

Japan

**Andre Okanobo**

Brazil

**Ólöf Olafsdottir**

Iceland

**J. Nwando Olayiwola**

USA

**UchechUKwu Osuagwu**

Australia

**Yoko Ozawa**

Japan

**Engin Bilge Ozgurhan**

Turkey

**Mark Packer**

USA

**Howard Palte**

USA

**Chen-Wei Pan**

China

**Eleni Papageorgiou**

UK

**George Papaliodis**

USA

**Chan Kee Park**

South Korea

**Choul Yong Park**

South Korea

**Sung Who Park**

South Korea

**Katrina Parker**

USA

**Salvador Pastor-Idoate**

Spain

**Sangita Patel**

USA

**Vanita Pathak Ray**

India

**Ushasree Pattamatta**

Australia

**Jayter Paula**

Brazil

**Michael Pavlica**

USA

**Gary Peh**

Singapore

**Enrico Peiretti**

Italy

**Gökhan Pekel**

Turkey

**Prakash Peralam Yegneswaran**

India

**Pablo Pérez-Merino**

Spain

**Henry Perry**

USA

**Simon Petersen-Jones**

USA

**John Phillips**

New Zealand

**Francesco Pichi**

Italy

**Jose Pinto**

Argentina

**Dilara Pirhan**

Turkey

**Amir Pirouzian**

USA

**Brigitte Pittet-Cuenod**

Switzerland

**Ana Belen Plaza**

Spain

**Eugenie Poh**

Singapore

**Lalitha Prajna**

India

**Gaurav Prakash**

United Arab Emirates

**Frank Proudlock**

UK

**Claudio Punzo**

USA

**Dania Qatarneh**

Australia

**Hong Qi**

China

**Kunliang Qiu**

China

**Mary Qiu**

USA

**Luciano Quaranta**

Italy

**Francesco Quaranta Leoni**

Italy

**Mansur Rabiu**

Nigeria

**Mohammad Taher Rajabi**

Iran

**Rajesh Rajagopalan**

Singapore

**Raju Rajala**

USA

**Vasant Raman**

UK

**Aparna Ramasubramanian**

USA

**Pradeep Ramulu**

USA

**Padmaja Kumari Rani**

India

**Aparna Rao**

India

**Dejan M. Rašić**

Serbia

**Rinki Ratnapriya**

USA

**Kimberly Reed**

USA

**Cornelius Rene**

UK

**Lixing Reneker**

USA

**Joanna Reszeć**

Poland

**Amanda Rey**

Spain

**Flavio Rezende**

Canada

**Seungsoo Rho**

South Korea

**Andri Riau**

Singapore

**Lucas Ricci**

Brazil

**Ivano Riva**

Italy

**Syed Rizvi**

India

**Gloria Roberti**

Italy

**Daniel Roberts**

USA

**Shisong Rong**

Hong Kong

**Maged Roshdy**

Egypt

**Luca Rossetti**

Italy

**Avik Roy**

India

**Sanhita Roy**

India

**Sayon Roy**

USA

**Jos Rozema**

Belgium

**Eliana Rulli**

Italy

**Kai Saariniemi**

Finland

**li Osman Saatci**

Turkey

**Sergio Saccà**

Italy

**Mahipal Sachdev**

India

**Mohammad Sadiq**

USA

**Manuel Saenz-De-Viteri**

Spain

**Masaaki Saito**

Japan

**Pavandeep Singh Sandhu**

UK

**Srinivasan Sanjay**

Singapore

**Muthupandian Saravanan**

Ethiopia

**Giacomo Savini**

Italy

**Ariel Schlaen**

Spain

**Leopold Schmetterer**

Austria

**Katrina Schmid**

Australia

**Heather Schmitt**

USA

**Lynn Schoenfield**

USA

**Stephen Schwartz**

USA

**Edina Schweighoffer**

UK

**Leonard Seibold**

USA

**Sirisha Senthil**

India

**Yasir Sepah**

USA

**Robert Sergott**

USA

**Ameet Shah**

UK

**Syed Shah**

USA

**Zaid Shalchi**

UK

**Siddesh Shambhu**

UK

**Robert Shanks**

USA

**S. Shantha**

India

**Emily Shao**

UK

**Heather Sheardown**

Canada

**Sethu Sheeladevi**

India

**Raneen Shehadeh Mashor**

Israel

**Yin Shen**

China

**Haihong Shi**

China

**Tomoaki Shiba**

Japan

**Rebecca Shields**

USA

**Rabia Shihada**

Israel

**Katsunori Shimada**

Japan

**Ari Shinojima**

Japan

**Jyoti Shrestha**

Nepal

**Ho Cheung (Anderson ) Shum**

Hong Kong

**Damian Siedlecki**

Poland

**Eric Sigler**

USA

**Ronald H. Silverman**

USA

**Huseyin Simavli**

Turkey

**Matthew Simunovic**

UK

**Michael Singer**

USA

**Eric Singman**

USA

**Sobha Sivaprasad**

UK

**Simon Skalicky**

UK

**Dimitra Skondra**

USA

**Wendy M Smith**

USA

**John Somner**

UK

**Kaz Soong**

USA

**Luigina Sorbara**

Canada

**Leopoldo Spadea**

Italy

**Oriel Spierer**

Israel

**Alex Spratt**

USA

**Sandra Spurr-Michaud**

USA

**Magi Sque**

UK

**Prema Sriram**

Australia

**Erin Stahl**

USA

**Miles Stanford**

UK

**David Steel**

UK

**John Stephenson**

UK

**Jay Stewart**

USA

**Gregory Stoddard**

USA

**Rupert Wolfgang Strauss**

USA

**Veit Sturm**

Switzerland

**Kaiming Su**

China

**Guanfang Su**

China

**Lakshman Subbaraman**

Canada

**Aditya Sudhalkar**

India

**Alan Sugar**

USA

**Michelle Sun**

Australia

**Young Sun**

USA

**Shivalingappa Swamynathan**

USA

**Mark Swanson**

USA

**Celine Sys**

UK

**Christopher Ta**

USA

**Audrey Talley Rostov**

USA

**Petrina Tan**

Singapore

**NaoyUKi Tanimoto**

Germany

**Angelo P. Tanna**

USA

**Jeremiah Tao**

USA

**Andrew Taylor**

USA

**Alejandro Tello**

Colombia

**Stephen Teoh**

Singapore

**Miguel Teus**

Spain

**Margarita Todorova**

Switzerland

**Mustafa Toker**

Turkey

**Ebru Toker**

Turkey

**Javier Tomás-Juan**

Spain

**Antonio Toso**

Italy

**Yuksel Totan**

Turkey

**Sharon Tow**

Singapore

**Chieh-Chih Tsai**

Taiwan

**Yeou-Ping Tsao**

Taiwan

**Michael Tsatsos**

UK

**Kiran Turaka**

USA

**Fatih Turkcu**

Turkey

**Phillip Turnbull**

New Zealand

**Argyrios Tzamalis**

Greece

**Takashi Ueta**

Japan

**F. Ussa**

USA

**Alejandra Valenzuela**

USA

**Tom Van Den Berg**

Netherlands

**Suzanne Van Landingham**

USA

**Gregory Van Stavern**

USA

**Lourens Van Zyl**

Australia

**Jean Vaudaux**

Switzerland

**Demetrios Vavvas**

USA

**Ramesh Ve**

India

**Federico Velez**

USA

**Rengaraj Venkatesh**

India

**Lingam Vijaya**

India

**Edoardo Villani**

Italy

**Guadalupe Villarreal Jr.**

USA

**Catherine Viner**

UK

**Gianni Virgili**

Italy

**Michael Volkert**

USA

**Graham Wallace**

UK

**Yan Wang**

China

**I-Jong Wang**

Taiwan

**An-Guor Wang**

Taiwan

**Gaofeng Wang**

USA

**Mengmeng Wang**

China

**Zhongxiao Wang**

USA

**Yi Wei**

USA

**Derek Welsbie**

USA

**Adam Wenick**

USA

**Eric Wieben**

USA

**Helmut Wilhelm**

Germany

**Cathy Williams**

UK

**David Williams**

UK

**Pete Williams**

USA

**Kimberly Winges**

USA

**Meraf Wolle**

USA

**Ian Wong**

Hong Kong

**Elizabeth Wong**

Singapore

**Wolf Wonneberger**

Sweden

**Se Joon Woo**

South Korea

**Emily Woodman**

Australia

**Fasika Woreta**

USA

**Yuan Wu**

China

**Jason CheUK-Sing Yam**

Hong Kong

**SUK-Woo Yang**

South Korea

**Chung-May Yang**

Taiwan

**Yit Yang**

UK

**Hui Yang**

China

**Yufeng Yao**

China

**Ke Yao**

China

**Yu-Feng Yao**

China

**Yasemin Yavuz**

Turkey

**Volkan Yaylali**

Turkey

**Tun K Yeo**

Singapore

**Zheng Qin Yin**

China

**Efdal Yoeruek**

Germany

**David Yoo**

USA

**Kyung Chul Yoon**

South Korea

**Sam Young Yoon**

South Korea

**Kim Yoon-Duck**

South Korea

**Yosanan Yospaiboon**

Thailand

**Honghua Yu**

China

**Minbin Yu**

China

**Murat Yuksel**

Turkey

**Patrick Yu-Wai-Man**

UK

**Leandro Zacharias**

Brazil

**Masahiro Zako**

Japan

**Juan Carlos Zenteno**

Mexico

**Xiaojun Zhang**

China

**Xiongze Zhang**

China

**Yuhua Zhang**

USA

**Shaoru Zhang**

China

**Xiulan Zhang**

China

**Chengxin Zhou**

USA

**Shiyou Zhou**

China

**Meidong Zhu**

Australia

**Mohammed Ziaei**

UK

**Noel Ziebarth**

USA

**Haidong Zou**

China

